# Urban Shrinkage and Depressive Symptoms Among Older Adults in China: Rural Burden and Threshold Effects

**DOI:** 10.1093/geroni/igaf122.4323

**Published:** 2025-12-31

**Authors:** Xinyue Wen, Fernando DePaolis

**Affiliations:** Middlebury College, Monterey, California, United States; Middlebury Institute of International Studies at Monterey, Monterey, California, United States

## Abstract

Urban shrinkage is rising globally, yet its mental-health implications for older adults—and how they differ by urban–rural context—remain unclear. Using four waves of the China Health and Retirement Longitudinal Study (CHARLS; unbalanced panel of 19,126 adults; 38,366 person-waves) linked to city-level population change, we examined depressive symptoms in later life. City-level data were defined at the prefecture level, encompassing both urban districts and rural counties; “urban/rural” denotes respondents’ residence. Individual fixed-effects models with time-varying covariates (age, gender, income, education, marital status) and wave controls revealed a robust gradient: shrinking rural > non-shrinking rural > shrinking urban > non-shrinking urban, indicating that the depressive-symptom burden is concentrated in shrinking rural contexts rather than uniformly across “shrinking cities.” To probe nonlinearity, we estimated segmented (piecewise linear) regressions of depressive symptoms on the continuous population-change rate, overall and stratified by residence, with the same covariate adjustment. Breakpoint analysis identified an adjusted overall threshold at − 12.0% population change (95% CI: −15.7%, −8.3%), beyond which depressive symptoms rose sharply. In rural areas, the threshold was −10.7% (95% CI: −13.6%, −7.8%); in urban areas, the estimated breakpoint was −5.7% (95% CI: −18.1%, 6.6%) and not statistically distinct. Findings highlight urban shrinkage as a place-based determinant of mental-health inequality in aging, with severe demographic decline disproportionately elevating depressive symptoms among rural older adults. As shrinkage becomes a global reality, strategies for mental health and aging should move beyond growth-oriented models toward targeted, place-sensitive interventions that bolster services, social support, and infrastructure in shrinking rural prefectures.

